# Sex-biased expression of selected chromosome x-linked microRNAs with potent regulatory effect on the inflammatory response in children with cystic fibrosis: A preliminary pilot investigation

**DOI:** 10.3389/fimmu.2023.1114239

**Published:** 2023-04-03

**Authors:** Maud Deny, Alexandros Popotas, Laurence Hanssens, Nicolas Lefèvre, Luis Alexis Arroba Nuñez, Ghislaine Simo Ouafo, Francis Corazza, Georges Casimir, Mustapha Chamekh

**Affiliations:** ^1^ Inflammation Unit, Laboratory of Pediatric Research, Faculty of Medicine, Université Libre de Bruxelles (ULB), Brussels, Belgium; ^2^ Université Libre de Bruxelles (ULB) Center for Research in Immunology (U-CRI), Brussels, Belgium; ^3^ Institut de Mucoviscidose – Unité Pédiatrique, Hôpital Universitaire des Enfants Reine Fabiola, Université Libre de Bruxelles (ULB), Brussels, Belgium; ^4^ Laboratoire de Médecine Translationnelle, Centre Hospitalier Universitaire Brugmann, Université Libre de Bruxelles (ULB), Brussels, Belgium

**Keywords:** cystic fibrosis, miRNA- microRNA, sex, inflammation, X chromosome (human)

## Abstract

Previous studies have reported sex disparity in cystic fibrosis (CF) disease, with females experiencing more pulmonary exacerbations and frequent microbial infections resulting in shorter survival expectancy. This concerns both pubertal and prepubertal females, which is in support to the prominent role of gene dosage rather than the hormonal status. The underlying mechanisms are still poorly understood. The X chromosome codes for a large number of micro-RNAs (miRNAs) that play a crucial role in the post-transcriptional regulation of several genes involved in various biological processes, including inflammation. However, their level of expression in CF males and females has not been sufficiently explored. In this study, we compared in male and female CF patients the expression of selected X-linked miRNAs involved in inflammatory processes. Cytokine and chemokine profiles were also evaluated at both protein and transcript levels and cross-analyzed with the miRNA expression levels. We observed increased expression of miR-223-3p, miR-106a-5p, miR-221-3p and miR-502-5p in CF patients compared to healthy controls. Interestingly, the overexpression of miR-221-3p was found to be significantly higher in CF girls than in CF boys and this correlates positively with IL-1β. Moreover, we found a trend toward lower expression in CF girls than in CF boys of suppressor of cytokine signaling 1 (SOCS1) and the ubiquitin-editing enzyme PDLIM2, two mRNA targets of miR-221-3p that are known to inhibit the NF-κB pathway. Collectively, this clinical study highlights a sex-bias in X-linked miR-221-3p expression in blood cells and its potential contribution to sustaining a higher inflammatory response in CF girls.

## Introduction

Cystic fibrosis (CF) is a monogenic disease caused by the mutation of cystic fibrosis transmembrane conductance regulator (CFTR) gene, an anion channel-protein coding sequence that plays a crucial role in the airway surface liquid homeostasis. CFTR dysfunction leads to an imbalance between Cl- secretion and Na+ uptake, resulting in airway surface dehydration and impaired mucociliary clearance (1, 2). A chronic inflammation within the lung is the hallmark of the disease, with exacerbation episodes linked to bacterial infection by opportunistic pathogens such as *Staphylococcus aureus* and *Pseudomonas aeruginosa*, leading to progressive airflow obstruction, tissue destruction and ultimately decreased respiratory function (1, 2).

We and others have reported sex-differences in the course of CF, with females experiencing more severe inflammation and exhibiting poorer prognosis compared to males (3–6). This sex disparity in CF exists despite the progress made in the early diagnosis and in the close therapeutic monitoring over the last years (6–10). Currently, the underlying mechanisms are poorly understood. Sex hormones and X-linked sexual genetic architecture have been proposed as potential factors (11–13). The essential role of the X chromosome is supported by the finding that such sex-differences in CF outcomes are also observed in young prepubertal children. Indeed, it has been shown that prepubertal girls have a significantly higher recruitment of neutrophils within the lungs and develop more episodes of infection than boys (3).

Interestingly, genomic distribution analysis revealed a high density of micro-RNAs (miRNAs) on the X chromosome (14). miRNAs are small non-coding RNA sequences of ±22 nucleotides that regulate gene expression at post-transcriptional level by repressing translation of specific mRNA targets through complementary pairing with their 3’ untranslated regions. Growing evidence points to their potential role in chronic inflammatory diseases impacting lung tissues, such as in CF (15–21). Indeed, studies have shown that CF patients have a dysregulation in the expression of number of miRNAs, including those targeting the mutated CFTR transporter or its derivatives, such as miR-145 or miR-494 (18, 22), or those involved in inflammatory pathology, such as the miR-126, miR-17 or miR-155 (23–25). Other studies showed a significant difference in the expression of X-linked miRNAs in monocytes from CF patients compared to non-CF patients (26). However, few reports investigated whether biological sex could impact miRNAs expression profiling in CF patients. Using miRNA array analysis of the plasma from six CF male and six CF female patients, Mooney et al. reported differential expression of two circulating miRNAs, miR-885-5p and miR-193a-5p, between girls and boys (27). Both miRNAs are not localized on the X chromosome and have no apparent role in the inflammatory process. Of note, females display mosaic cells expressing two X-linked gene alleles and one of the X chromosomes is randomly inactivated early in embryogenesis to ensure the compensation dosage. Yet, about 15-23% of human X-chromosome-linked genes escape the inactivation with some variability between tissues and individuals (28, 29). This may result in differential expression of X-linked genes between males and females. In this study, we asked whether blood leukocytes from male and female CF patients exhibit a differential expression profile of X-linked miRNAs with potent impact on the inflammatory process. We focused on miRNAs that have been shown to be dysregulated in the airways of CF patients compared to healthy individuals (23) and for which there is accumulating evidence of their direct or indirect role in the regulation of inflammation.

## Materials and methods

### Ethics statement

All blood samples were obtained from control subjects and CF patients at the Queen Fabiola University Children’s Hospital (HUDERF) in Brussels, Belgium. Written consent was obtained from the children’s parents or legal guardians. The protocols were approved by the hospital ethics committee of the Brugmann University Hospital (CE 2016/162).

### Characteristics of patients

29 CF patients (15 males and 14 females) and 20 healthy controls (10 males and 10 females) were recruited. The patients were diagnosed using a sweat test and checked for CFTR gene mutation. The different characteristics of the patients are presented in **Table 1**.

**Table 1:** Characteristics of CF patients and healthy controls enrolled in the study.

Characteristics	CF	Healthy
Number of samples	29	20
Sex, male/female (%men)	15/14 (51.7)	10/10 (50)
Age (years), median (IQR) male/female (p-value)	7.875 (10,92) 10.58 (10,44)/6.54 (6,08) (0,1616)	6.75 (4,71) 4.89 (4,89)/5.88 (3,94) (0,8405)
Severe mutations (class I, II and III), n (%) male/female	22 (73.33) 13/9	NA
pancreatic insufficiency, n (%) male/female	22 (73.33) 13/9	NA
CRP (mg/L), median (IQR) Male/female (p-value)	0,6 (2,6) 1,2 (2,6)/0.5 (2,33) (0.7261)	NA
ESR (mm/h), median (IQR) Male/female (p-value)	7,5 (7,5) 7 (8)/9 (6) (0.7415)	NA
FEV1 (%), median (IQR) Male/female (p-value)	84 (15,75) 82 (17,5)/85 (9) (0.8671)	NA
Leukocytes (*10^3^/µL), median (IQR) Male/female (p-value)	8810 (4060) 8590 (4175)/9145 (3000) (0.9236)	NA
Neutrophiles (*10^3^/µL), median (IQR) Male/female (p-value)	4350 (1690) 4350 (1680)/4285 (1955) (0,7800)	NA
Monocytes (*10^3^/µL), median (IQR) Male/female (p-value)	640 (280) 640 (410)/630 (147,5) (0,6128)	NA
Lymphocytes (*10^3^/µL), median (IQR) Male/female (p-value)	3050 (1760) 2840 (2670)/3450 (1250) (0,7148)	NA

The biological parameters were performed during the annual check-up and the FEV1 was taken during the consultation. p-values were calculated between males and females with the Mann-Whitney test. IQR: interquartile range, CRP: C-reactive protein, ESR: Erythrocyte sedimentation rate, FEV1: the first second of forced expiration.

### Sample processing

Blood samples were collected in EDTA tubes. Blood from CF patients and healthy controls was processed within 4 hours of collection to extract plasma and leukocytes. After extraction from plasma, blood cells were treated with the erythrolysis buffer and leucocytes were resuspended in Trizol (Qiagen). The plasma and leucocytes were stored at -80°C until use.

### Detection of inflammatory cytokines in plasma

The quantification of inflammatory mediator levels in the plasma was performed by Luminex Multiplex Assays (ThermoFischer, EPX210-1585 0-901) according to the manufacturer’s instructions. Plate reading and quantification were performed using MAGPIX™ Instrument System (Invitrogen), and the results were analyzed using the ProcartaPlex™ program (ThermoFischer). In most samples, cytokine levels were above detection limits. The extrapolation was made for samples that were below the detection limit.

### Total RNA extraction and miRNAs and mRNAs expression analysis

Total RNA was isolated using the miRNeasy mini kit (Qiagen) following the manufacturer’s instructions from leukocytes samples. RNA concentrations were assessed using the NanoVue Plus spectrophotometer (Biochrom). The reverse transcription of total RNA (including miRNAs) was performed with the miScript II RT Kit (Qiagen) following the manufacturer’s instructions. qRT-PCR was performed with the miScript SYBR Green PCR Kit (Qiagen) according to standard protocols. We used the small-nucleolar RNA C/D box 95 (SNORD95) as miRNA internal control and two house-keeping genes (GAPDH and beta-actin) for mRNA expression analysis. Reactions with no-template and no-reverse transcriptase were performed to unsure negative controls. The specificity of each primer pairs was further verified by controlling the melt curve profile. Relative quantification of miRNAs expression was calculated using 2^−ΔΔCt^ method; the fold change differences were relative to sex-matched healthy controls. The primers used in this study are listed in **Table 2**.

**Table 2:** Oligonucleotide primers used for miRNA and mRNA qRT-PCR analysis.

GENE	Forward Primer Sequence (5’ → 3’) or name of primer	Reverse Primer Sequence (5’ → 3’) or name of primer	
miR-223-3p	TGT-CAG-TTT-GTC-AAA-TAC-CCC-A	miScript Universal Primer (Qiagen)
miR-106a-5p	AAA-AGT-GCT-TAC-AGT-GCA-GGT-AG	miScript Universal Primer (Qiagen)
miR-221-3p	AGC-TAC-ATT-GTC-TGC-TGG-GTT-TC	miScript Universal Primer (Qiagen)
miR-502-5p	ATC-CTT-GCT-ATC-TGG-GTG-CTA	miScript Universal Primer (Qiagen)
miR-374a-5p	TTA-TAA-TAC-AAC-CTG-ATA-AGT-G	miScript Universal Primer (Qiagen)
miR-20b-5p	CAA-AGT-GCT-CAT-AGT-GCA-GGT-AG	miScript Universal Primer (Qiagen)
miR-532-5p	CAT-GCC-TTG-AGT-GTA-GGA-CCG-T	miScript Universal Primer (Qiagen)
SNORD95	GCG-GTG-ATG-ACC-CCA-ACA-T	miScript Universal Primer (Qiagen)
IL-8	GAC-CAC-ACT-GCG-CCA-ACA-C	CTT-CTC-CAC-AAC-CCT-CTG-CAC
IL-6	AAT-TCG-GTA-CAT-CCT-CGA-CGG	GGT-TGT-TTT-CTG-CCA-GTG-CC
IL-1β	ACA-GAT-GAA-GTG-CTC-CTT-CCA	GTC-GGA-GAT-TCG-TAG-CTG-GAT
TNFα	CCC-AGG-GAC-CTC-TCT-CTA	ATG-GGC-TAC-AGG-CTT-GTC-ACT
A20	CGT-CCA-GGT-TCC-AGA-ACA-CCA-TTC	TGC-GCT-GGC-TCG-ATC-TCA-GTT-G
PDLIM2	GAG-AAG-TGC-AGT-ACC-AGC-ATC-G	GCA-TCT-TCA-GGT-TCA-GCC-CAC-A
GAPDH	GTC-GCT-GTT-GAA-GTC-AGA-GG	GAA-ACT-GTG-GCG-TGA-TGG
β-actin	GGA-TGC-AGA-AGG-AGA-TCA-CTG	CGA-TCC-ACA-CGG-AGT-ACT-TG

### Statistical analysis

We used the non-parametric Mann-Whitney test for independent groups to compare males and females. Correlations were tested using the non-parametric Spearman test. Bonferroni correction was applied when needed. Differences were considered statistically significant with p (two-tailed) <0.05 using Prism 8.0 statistical software (GraphPad, San Diego, California), with *, **, and *** symbols respectively signify p <0.05, p <0.01, and p <0.001.

## Results

### Comparative analysis of the expression of inflammatory mediators

A persistent and damaging chronic inflammation is the hallmark of CF pathology. To evaluate the clinical status of the patients, we analyzed various inflammatory parameters. As shown in **Table 1**, patients included in this study have a relatively stable clinical situation with low levels of C-reactive protein (CRP), erythrocyte sedimentation rate (ESR), normal blood cell composition, and a mild to moderate pulmonary phenotype as measured by first second of forced expiration (FEV1). Of note, no sex differences were observed for these parameters (**Table 1**).

**Figure 1:**
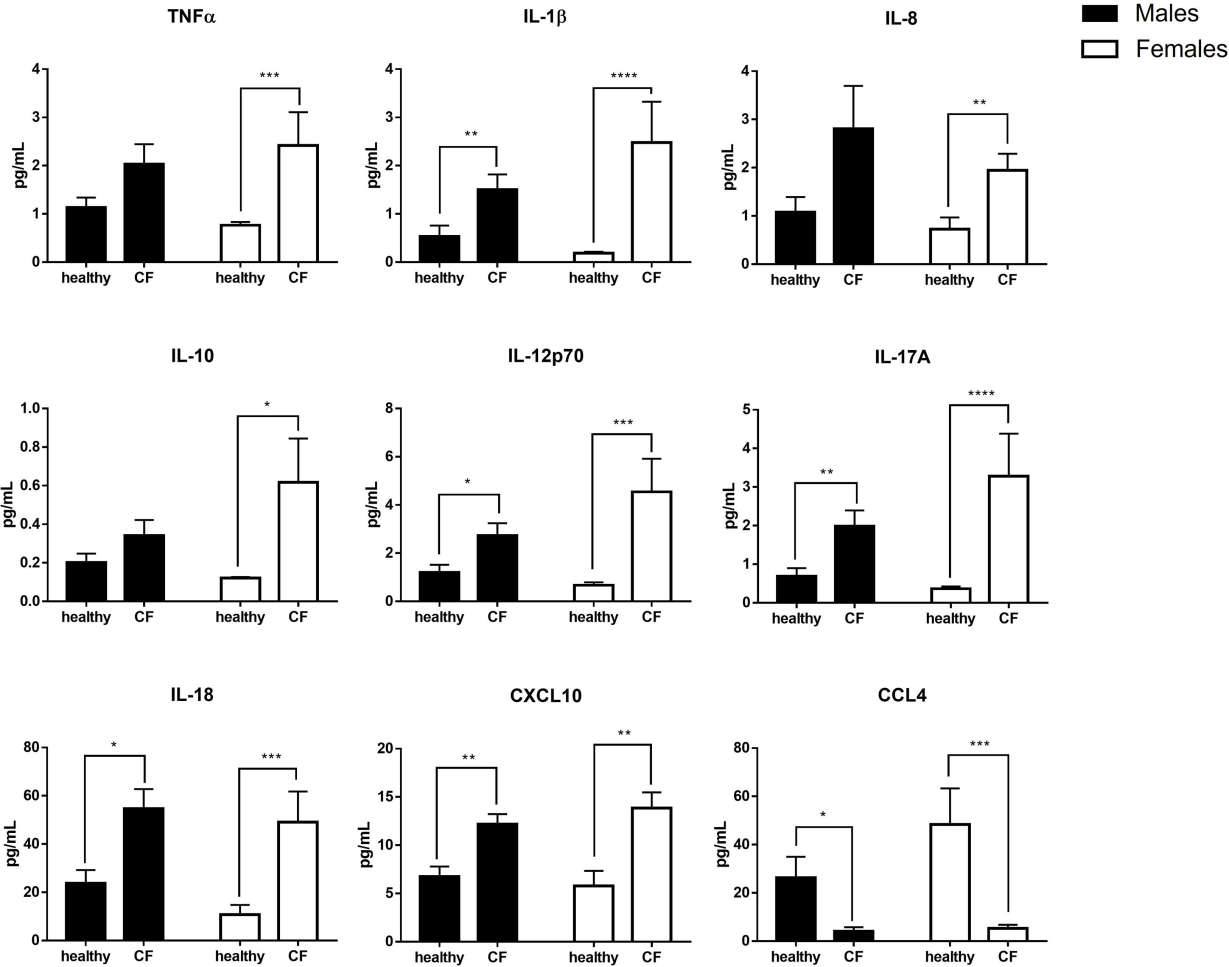
Sex-based analysis of the production of pro- and anti-inflammatory mediators in the plasma of CF patients (n=29; 15 males and 14 females) and healthy controls (n=20; 10males and 10 females). Graphs show the mean ± SEM. *p<0,05; **p<0,01; ***p<0,001: ****p<0,0001 (Mann Whitney U test).

To further analyze the inflammatory state of CF patients, we also evaluated the balance of circulating pro- and anti-inflammatory mediators (TNFα, IL-1β, IL-6, IL-8, IL-10, IL-12p70, IL-17A, IL-18, CCL2, CCL3, CXCL10 and CCL4) in plasma. Compared with healthy controls, CF patients had higher amount of most cytokine and chemokine mediators, with the exception of CCL4 which was significantly lower. We found no obvious difference between CF boys and girls, although we noticed a marked production of TNFα, IL-1β, IL-8, IL-10, IL-12p70, IL-17A, and CXCL10 in CF females than CF males when compared to their matched controls (**Figure 1**).

**Figure 2:**
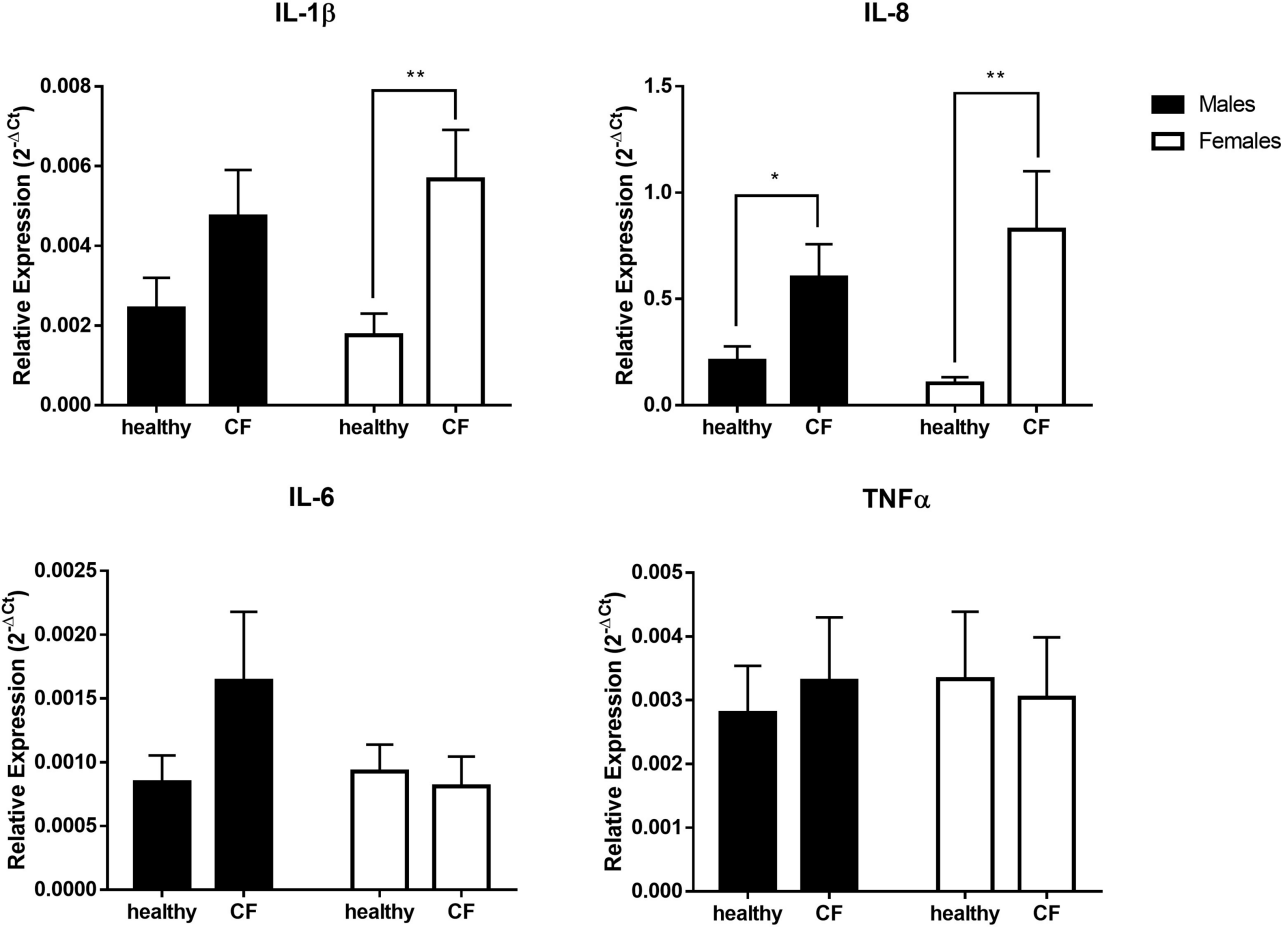
Sex-based assessment of inflammatory cytokines expression in blood leukocytes from male healthy controls (n=10) versus male CF patients (n=15) and female healthy controls (n=10) versus female CF patients (n=14). The values were normalized with two internal controls, GAPDH and β-actin. Graphs show the mean ± SEM. *p<0,05; **p<0,01 (Mann Whitney U test).

In parallel, we evaluated by qRT-PCR the expression of major inflammatory cytokine transcripts. As shown in **Figure 2**, the level of expression of IL-1β is significantly higher only in CF females compared to control, while the expression IL-8 increased in both CF males and females compared to sex-matched controls. We found, however, no significant difference for IL-6 and TNFα. Overall, although CF patients enrolled in this study exhibited a relatively stable clinical state, these results indicate an upregulation of several inflammatory mediators that might impact the physiopathology.

### X-linked miRNA expression profile in blood leukocytes from CF male and female patients

**Figure 3:**
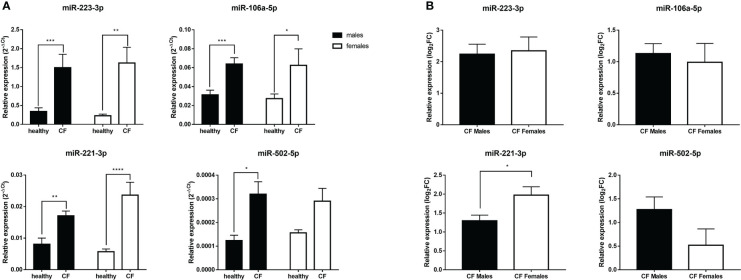
**(A)** Sex-based comparative study of the expression of X-linked miR-221-3p, miR-502-5p, miR-223-3p and miR-106a-5p in blood leukocytes of male healthy controls (n=10) versus CF males (n=15) and female healthy controls (n=10) versus CF female patients (n=14). The values (2^-ΔCT^) were normalized with the internal control SNORD95. **(B)** Sex-based comparative study of fold change values (log_2_(2^-ΔΔCT^)) of X-linked miR-221-3p, miR-502-5p, miR-223-3p and miR-106a-5p expression in blood leukocytes between CF males (n=15) and CF females (n=14). The fold change values (log_2_(2^-ΔΔCT^)) are relative to those from healthy male or female controls (n=10/group). Graphs show the mean ± SEM. *p<0,05; **p<0,01; ***p<0,001; ****p<0,0001 (Mann Whitney U test).

Next, we sought to investigate by qRT-PCR the relative expression of a panel of X-linked miRNAs in blood leucocytes. Specifically, we wished to explore the expression profile of miR-223-3p, miR-106a-5p, miR-221-3p, miR-502-5p, miR-374a-5p, miR-20b-5p and miR-532-5p given their modulatory role, shown previously in different inflammatory settings (30–36), and also because their expression was found to be dysregulated in CF airway epithelial cells (23). We first analyzed all the patients regardless of sex. The expression level of miR-374a-5p, miR-20b-5p and miR-532-5p did not significantly change in CF patients compared to controls in either boys or girls (data not shown). However, different profile was obtained for the other tested miRNAs. We found a significant increase in the expression of miR-223-3p, miR-106a-5p, and miR-221-3p in both CF males and females compared to their sex-matched controls (**Figure 3A**). The expression of miR-502-5p is significantly higher only in CF males compared to controls (**Figure 3A**). We also evaluated the magnitude of the relative expression of these miRNAs in males and females, by considering the fold changes to their matched controls. As shown **Figure 3B**, we found no significant difference for miR-502 between males and females, whereas the overexpression of miR-221-3p is significantly higher in CF females than in CF males.

**Figure 4:**
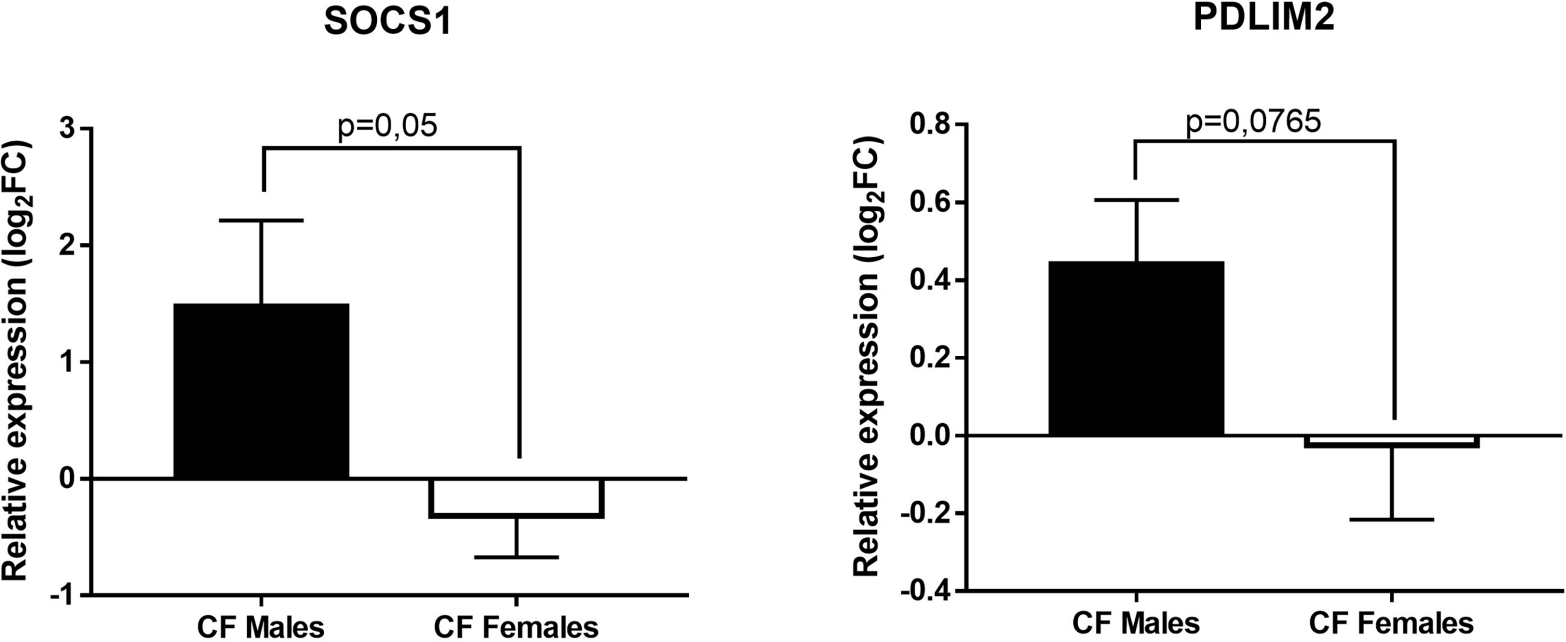
Sex-based comparative study of fold change values (log_2_(2^-ΔΔCT^)) of SOCS1 and PDLIM2 expression in blood leukocytes between CF males (n=15) and CF females (n=14). The fold change values (2^-ΔΔCT^) are relative to those from sex-matched healthy controls (n=10/group). Graphs show the mean ± SEM.

Next, we tested whether the overexpression of miR-221-3p in females could impact the expression of its known mRNA targets: A20 and PDLIM2, two ubiquitin-editing enzymes, and SOCS1 and SOCS3 that are inhibitors of JAK/STAT and NF-kB signaling pathways (32, 37–39). We also tested JAK3, a newly identified miR-221-3p target (40). The data of PDLIM2 and SOCS1 are shown in **Figure 4**. There is a substantial difference with a trend towards lower expression of SOCS1 (p=0,05) in CF females compared to CF males. Similar trend is observed for PDLIM2, while no obvious difference could be seen for A20, SOCS3 and JAK3 (data not shown)

It has been reported that sex difference in inflammatory response in CF can be observed in infants under 8 years of age where the level of sex hormones is usually very low (3). We analyzed data in the subgroup of prepubertal infants (age <8 years) to see if there were any gender differences. As for the entire cohort, we observed that miR-221-3p is overexpressed in CF prepubertal females compared to males with the same trends of lower expression of the PDLIM2 and SOCS1 mRNA targets (**Figure S1**). Although the number of patients remains small, these results suggest that sex hormones are less likely to play a major role.

### The overexpression of miR-221-3p in CF patients correlates positively with the upregulation of IL-1β transcript

**Figure 5:**
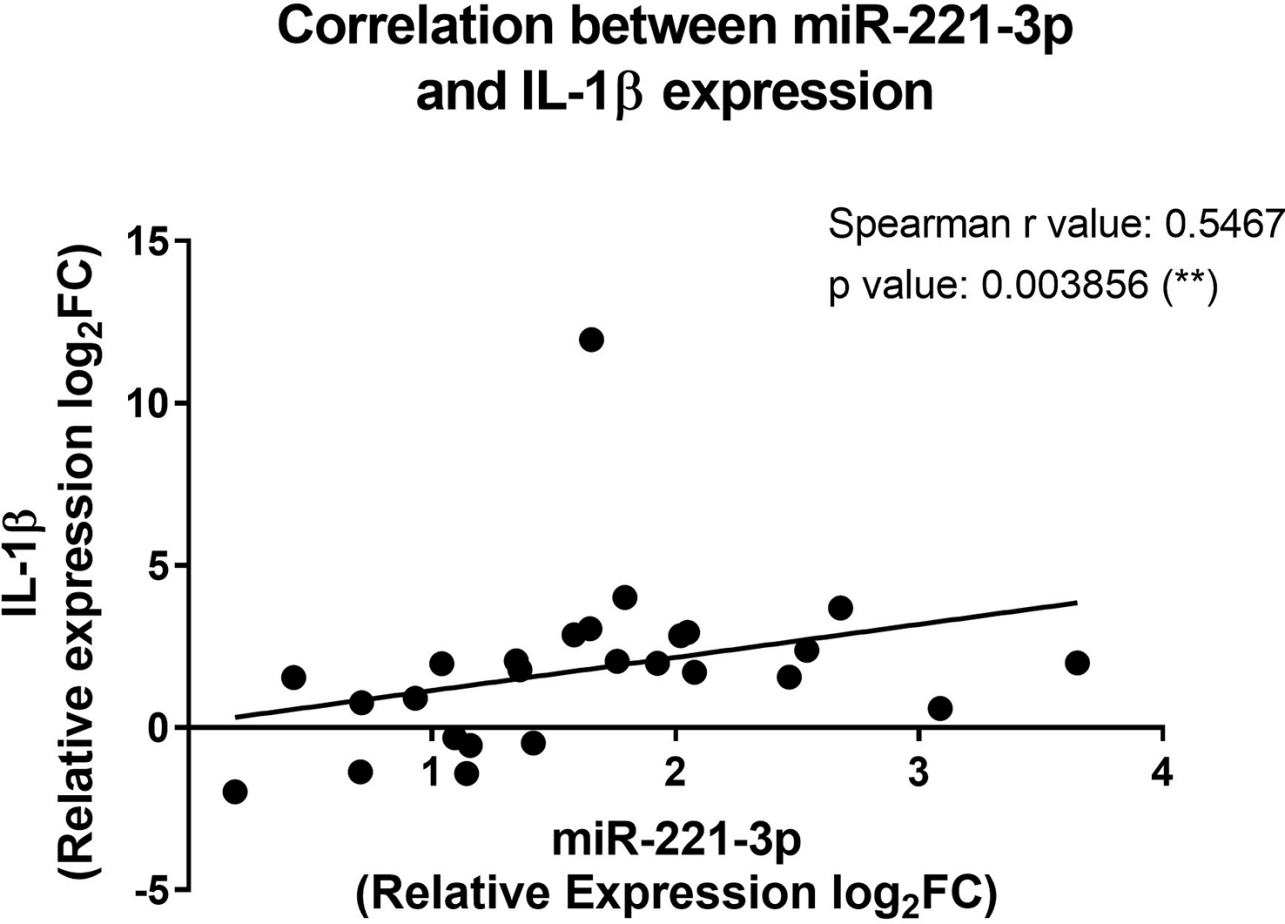
Correlation between the miR-221-3p and the IL-1β expression in blood leukocytes from CF patients. The fold change values (2^-ΔΔCT^) are relative to those from healthy controls (n=10/group). **p<0,01 (Spearman test).

To see whether the expression profile of miRNAs correlates with the inflammatory state of CF patients, we cross-analyzed the expression level of each miRNA with all tested cytokines and chemokines, both at protein and transcript levels. The only significant positive correlation was observed between miR-221-3p and IL-1β transcript (**Figure 5**), a finding consistent with the pro-inflammatory role of this microRNA. This seems also true when considering only prepubertal (age <8 years) boys and girls (**Figure S2**), suggesting that sex hormones may not have a great impact.

## Discussion

It is well documented that the biological sex is a contributing factor in the susceptibility to CF, with a better prognosis for boys compared to girls. However, there is paucity of data related to molecular markers that could be relevant to the mechanisms at work. In this study, we examined the potential contribution of selected X-linked miRNAs that are involved in the regulation of the inflammatory process and whose aberrant expression has been described in CF patients.

Despite the overall higher production of pro-inflammatory cytokines in CF compared with controls, patients enrolled in this study exhibited a relatively stable clinical state with respect to clinical parameters routinely recorded during medical monitoring. Similar observations have been previously reported in human and experimental CF (41–45). Currently, better therapeutic care that significantly improves the outcome of the disease is progressing thanks to the newborn screening allowing an early diagnosis. While gender difference still exists in CF, significant advances in timely treatment and patient follow-up, have largely contributed to reduce the difference observed in the pathophysiological evolution and inflammatory phenotype between boys and girls. However, substantial differences between males and females could be detected at the transcript level, in particular using signal amplification methods such as qRT-PCR.

Regarding miRNAs, we observed a significant increase in the expression of miR-223-3p, miR-106a-5p, miR-502-5p and miR-221-3p in blood cells from CF patients compared with healthy controls. The miR-223-3p is highly expressed by myeloid cells and is particularly involved in the control of granulopoiesis (46). In addition, we and others have previously shown that miR-223-3p is an essential modulator of the inflammation through the regulation of neutrophil activity and macrophage polarization (47–52). The overexpression of miR-223-3p observed in blood leukocytes of CF patients is in line with previous studies describing its upregulation in CF endobronchial brush samples (23) as well as in a CFTR gene-mutated bronchial epithelial cell line (53). Further studies are needed to depict the functional role of miR-223-3p in the inflammatory pathogenesis of CF. On the other hand, the miR-106a-5p is known to be involved in the control of monocytopoiesis (54) and different studies suggested its anti-inflammatory potential (31, 55). In CF, the expression of miR-106a was shown to be downregulated in endobronchial brush samples (23). In contrast, we detected a significant expression of miR-106a in blood leukocytes in our CF cohort. This discrepancy could be explained by differences in the samples used (endobronchial brush versus blood leukocytes), in patient’s clinical status or in the experimental design related to data normalization. It is likely that the increase in the expression level of miR-106a in the blood cells of patients with cystic fibrosis can be considered as one of the compensatory mechanisms to limit excessive inflammation. On the other hand, few studies addressed the role of miR-502-5p in inflammation. Zhang G et al. reported it potential role in inhibiting the IL-1β-induced NF-κB pathway in chondrocytes by targeting TRAF2 (33). The contribution of miR-502-5p in the inflammatory signaling pathways in CF remains unclear.

Notably, this study revealed that miR-221-3p is significantly overexpressed in blood cells from CF females compared to CF males. Similar trends were obtained when comparing prepubertal CF patients, suggesting a non-prominent role of sex hormones. However, this requires a large cohort and hormone assay for a firm conclusion. Since no significant difference were observed in leukocyte count and blood cell composition between genders, the overexpression of miR-221-3p seen in females would most likely be attributed to a differential cell activation and/or gene inactivation escape occurring naturally in one of the X chromosomes. The miR-221 was mainly investigated in cancer where studies have shown its potential contribution to tumor progression through modulation of cell proliferation, invasion, and apoptosis (56). Moreover, its aberrant expression was reported in inflammatory diseases as well, like in rheumatoid arthritis, atherosclerosis and asthma (57, 58). In CF, miR-221-3p was shown to be overexpressed in CF airway epithelial cells (59). Interestingly, we found that the overexpression of miR-221-3p in CF blood cells is positively correlated with the expression of IL-1β. We also observed a trend towards lower expression in CF females of SOCS1 and PDLIM2, two mRNA targets of miR-221-3p. Of note, it would be interesting to investigate how miR-221-3p could impact the expression of its main targets at protein level as well. Although the causative link between miR-221 and IL-1β-related inflammatory profile within blood cells derived from CF patients remains unclair, this observational clinical study is in line with previous experimental studies supporting the pro-inflammatory role for miR-221-3p by promoting the activation of the NF-κB signaling pathway (32, 37–39, 60).

Our investigation addresses for the first time the potential contribution of X-linked miRNAs derived from CF blood leukocytes in the sex-bias of the inflammatory response. The study has some limitations. We cannot rule out the role of CFTR gene mutation type that may have different functional consequences, ranging from mild to a severe disease phenotype (61). As mentioned elsewhere, although our results do not appear to be affected when considering only prepubertal patients, the cohort remains too small to rule out a potential impact of sex hormones on miRNA expression (62, 63). It would be interesting to investigate, in future studies, a large homogenous cohort that includes not only low to moderate clinical phenotypes but also cases with pulmonary exacerbation to examine whether the gender difference in miRNA expression may have an important impact on the inflammatory process. Collectively, this pilot study suggests the potential contribution of X-linked pro-inflammatory miR-221-3p in the sex-bias of the inflammatory process and highlights the need to unravel its role in the pathophysiology of CF and determine how this may help optimize the personal follow-up of patients.

## Data availability statement

The original contributions presented in the study are included in the article/**Supplementary Material**. Further inquiries can be directed to the corresponding author.

## Ethics statement

The studies involving human participants were reviewed and approved by the hospital ethics committee of the Brugmann University Hospital (CE 2016/162). Written informed consent to participate in this study was provided by the participants’ legal guardian/next of kin.

## Author contributions

MD performed experiments, analyzed data, and wrote the first draft. AP patient recruitment and reviewed the manuscript. LH patient recruitment and reviewed the manuscript. NL patient recruitment and reviewed the manuscript. LAAN performed experiments. GSO performed experiments. FC reviewed the manuscript. GC reviewed the manuscript. MC supervised the work, analyzed data, and reviewed the manuscript. All authors contributed to the article and approved the submitted version.
