# Supplementary material for: Sex-biased expression of selected chromosome x-linked microRNAs with potent regulatory effect on the inflammatory response in children with cystic fibrosis: A preliminary pilot investigation
Source: Front Immunol. 2023 Apr 3;14:1114239. doi: 10.3389/fimmu.2023.1114239 (PMC10106689; doi:10.3389/fimmu.2023.1114239)
Supplement: Supplementary file 1 [file DataSheet_1.pdf]

Investigation of sex-biased expression of selected chromosome X-linked miRNAs with potent regulatory effect on the inflammatory response in children with cystic fibrosis

Maud Deny[1,2], Alexandros Popotas[1], Laurence Hanssens[3], Nicolas Lefèvre[1,3], Luis Alexis Arroba Nuñez[1,2], Ghislaine Simo Ouafo[1,2], Francis Corazza[4], Georges Casimir[1,3] and Mustapha Chamekh[1,2]*

[1] Inflammation Unit, Laboratory of Pediatric Research, Faculty of Medicine, Université Libre de Bruxelles (ULB), Brussels, Belgium.

[2] ULB Center for Research in Immunology (U-CRI), Brussels, Belgium.

[3] Institut de Mucoviscidose – Unité pédiatrique, Hôpital Universitaire des Enfants Reine Fabiola, Université libre de Bruxelles (ULB), Brussels, Belgium

[4] Laboratoire de Médecine Translationnelle, Centre Hospitalier Universitaire Brugmann, Université Libre de Bruxelles (ULB), Brussels, Belgium.

* Corresponding author: Mostafa.chamekh@ulb.be

Supplementary data

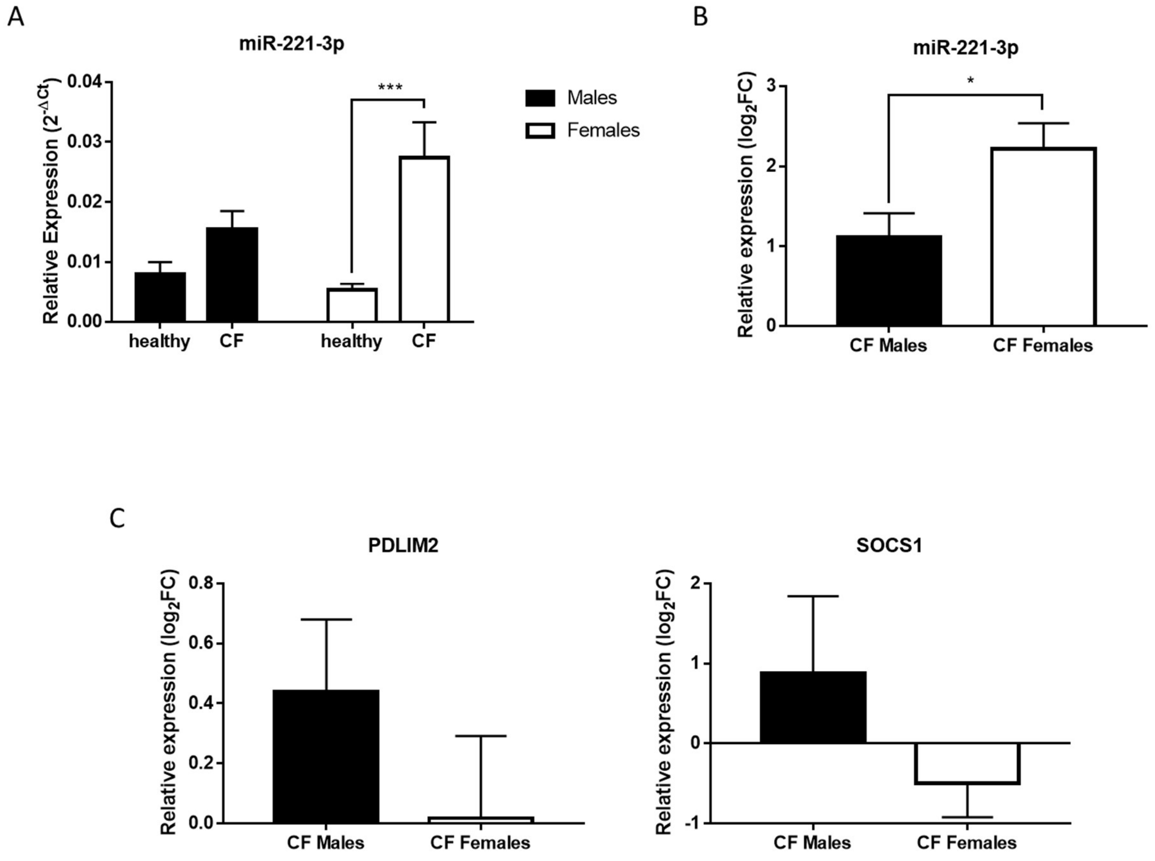

Figure S1. A. Sex-based analysis of miR-221-3p expression in blood leukocytes from prepubertal (<8 yr) male healthy controls (n=7) versus prepubertal (<8 yr) male CF patients (n=6) and from prepubertal (<8 yr) female healthy controls (n=6) versus prepubertal (<8 yr) female CF patients. The values ($2^{-\Delta CT}$) were normalized with the internal control SNORD95. **B.** The fold change values ($\log_2(2^{-\Delta\Delta CT})$) are relative to those from sex-matched healthy controls. **C.** Sex-based analysis of miR-221-3p target expression in blood leukocytes from prepubertal (<8 yr) male healthy controls (n=7) versus prepubertal

(<8 yr) male CF patients (n=6) and from prepubertal (<8 yr) female healthy controls (n=6) versus prepubertal (<8 yr) female CF patients (n=9). The results were normalized with two internal controls, GAPDH and β-actin. The fold change values ($\log_2(2^{-\Delta\Delta CT})$) are relative to those from sex-matched healthy controls. Graphs show the mean ±SEM. *p<0,05; ***p<0,001 (Mann Whitney U test).

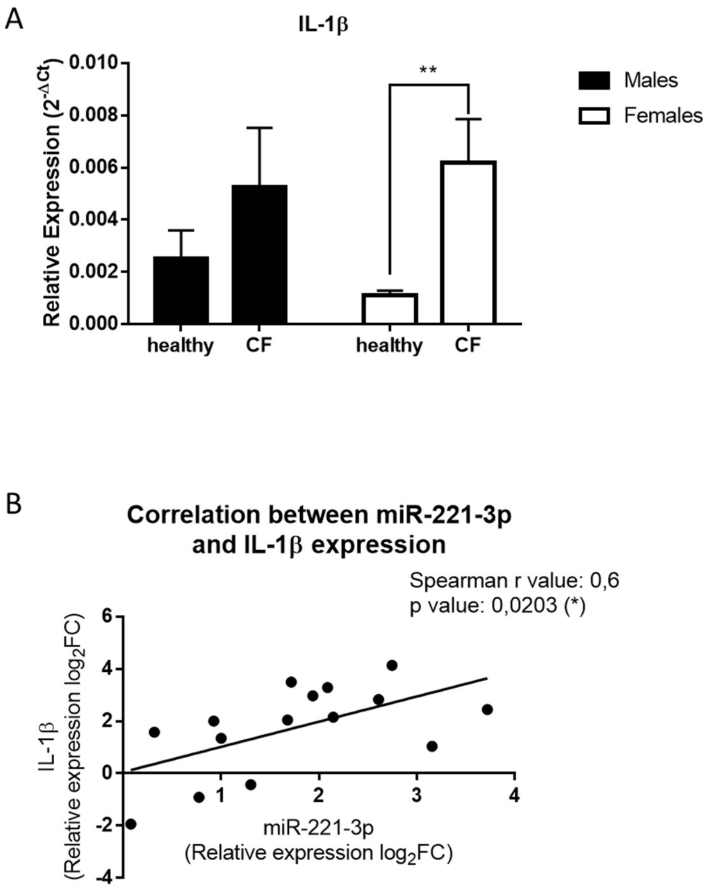

Figure S2. A. Sex-based assessment of IL-1β expression in blood leukocytes from prepubertal (<8 yr) male healthy controls (n=7) versus prepubertal (<8 yr) male CF patients (n=6) and from prepubertal (<8 yr) female healthy controls (n=6) versus prepubertal (<8 yr) female CF patients (n=9). The values ($2^{-\Delta CT}$) were normalized with two internal controls, GAPDH and β-actin. Graph shows the mean ±SEM. **p<0,01 (Mann Whitney U test). **B.** Correlation between the miR-221-3p and the IL-1β expression in blood leukocytes from prepubertal (<8yr) CF patients. *p<0,05 (Spearman test).
